# Estimating 24-hour urine phosphate excretion from spot urine

**DOI:** 10.1093/ckj/sfaf097

**Published:** 2025-04-10

**Authors:** Yongchao Li, Daniel G Fuster, Nasser A Dhayat, Harald Seeger, Alexander Ritter, Olivier Bonny, Gregoire Wuerzner, Thomas Ernandez, Stephan Segerer, Beat Roth, Isabel Rubio-Aliaga, Carsten A Wagner

**Affiliations:** Institute of Physiology, University of Zurich, Zurich, Switzerland; Department of Urology, Xiangya Hospital, Central South University, Changsha, China; National Clinical Research Center for Geriatric Disorders, Xiangya Hospital, Central South University, Changsha, China; National Center of Competence of Research NCCR Kidney.CH; Department of Nephrology and Hypertension, Inselspital, Bern University Hospital, University of Bern, Switzerland; Nephrology & Dialysis Care Center, B. Braun Medical Care AG, Hochfelden, Zurich, Switzerland; Division of Nephrology, University Hospital Zurich, Zurich, Switzerland; Institute for Nephrology and Dialysis, Cantonal Hospital Baden, Baden, Switzerland; Division of Nephrology, University Hospital Zurich, Zurich, Switzerland; Clinic for Nephrology and Transplantation Medicine, Cantonal Hospital St. Gallen, St. Gallen, Switzerland; National Center of Competence of Research NCCR Kidney.CH; Service of Nephrology, Fribourg State Hospital and University of Fribourg, Fribourg, Switzerland; Service of Nephrology and Hypertension, Lausanne University Hospital and University of Lausanne, Lausanne, Switzerland; Service of Nephrology and Hypertension, Lausanne University Hospital and University of Lausanne, Lausanne, Switzerland; Service of Nephrology, Geneva University Hospitals, Geneva, Switzerland; Division of Nephrology, Kantonsspital Aarau, Aarau, Switzerland; Department of Urology, Inselspital, University of Bern, Bern, Switzerland; Institute of Physiology, University of Zurich, Zurich, Switzerland; National Center of Competence of Research NCCR Kidney.CH; Institute of Physiology, University of Zurich, Zurich, Switzerland; National Center of Competence of Research NCCR Kidney.CH

**Keywords:** 24-hour urine phosphate, kidney disease, predictive equation, spot urine, the Swiss Kidney Stone Cohort

## Abstract

**Background:**

24-hour urinary phosphate excretion (24hUrP) is indicative of intestinal phosphate absorption in steady-state conditions. Nevertheless, 24-hour urine collections are cumbersome and error-prone. Previous studies suggested that spot urine phosphate (uPi) could serve as a practical substitute to predict 24hUrP, however, these data originated only from patients with chronic kidney disease. Here, we investigated the validity of predictive equations using spot urine parameters to assess 24hUrP in a cohort with normal kidney function (eGFR >60 ml/min per 1.73 m^2^) including 761 kidney stone patients and 207 non-kidney stone formers as assessed by low-dose CT scans, the Swiss Kidney Stone Cohort (SKSC).

**Methods:**

Published equations for 24hUrP were tested in our cohort and a novel predictive equation was developed. Pearson correlation coefficients and Bland–Altman plots were used to assess the relationship between spot uPi and spot urine creatinine (uCr) and 24hUrP. Additionally, forward multivariate analysis was performed to predict uPi excretion.

**Results:**

Previously published equations provided less accurate prediction of 24hUrP from spot urine. Log-transformed 24hUrP with log-transformed spot uPi and creatinine yielded the best model fit. In addition, inclusion of age, sex, and BMI significantly improved prediction of 24hUrP. Compared with spot uPi and uCr alone (*r*^2^ = 0.0561, *P* < .001) the new equation predicted 24hUrP (*r*^2^ = 0.1820, *P* < .001) more accurately.

**Conclusions:**

Here, we present a new equation for predicting 24hUrP from spot urine samples of individuals with normal kidney function. This model has a moderate ability to explain 24hUrP variance but has the strength to use only parameters routinely collected in clinical settings such as spot urinary phosphate and creatinine, sex, BMI, and age.

KEY LEARNING POINTS **What was known**:

- Previous studies suggested that spot urine phosphate (uPi) could serve as a practical and effective substitute to predict 24-hour urinary phosphate excretion (24hUrP).
- Different equations have been published to estimate 24hUrP from spot urine samples of patients with CKD but not individuals with normal kidney function.

**This study adds**:

- In a cohort of individuals with normal kidney function including kidney stone formers and non-kidney stone formers, the published formulas failed to predict 24hUrP.
- We present a new equation for predicting 24hUrP by incorporating spot urinary phosphate and creatinine, along with sex, BMI, and age.

**Potential impact**:

- This equation allows a simple and inexpensive estimation of 24hUrP from spot urine.
- The equation can be applied in participants with normal kidney function.

## INTRODUCTION

Urine analysis is an important component of the metabolic analysis of patients with suspected tubulopathies, some endocrinopathies, kidney stones, or other forms of kidney disease [1–3]. It may also be helpful for assessing nutritional intake of salt, phosphate, proteins of animal or plant origin, and acid or alkali equivalents [4–6]. Depending on the purpose of the urine analysis, spot urine or 24-hour urine collections may be required.

Analysis of urinary phosphate excretion can be helpful in disorders with nephrolithiasis, hypophosphatemia (e.g. hypophosphatemic rickets, post liver transplant), or disorders of abnormal bone mineralization. It is generally assumed that in healthy individuals in steady-state the 24-hour urine phosphorus excretion (24hUrP) reflects the daily amount of phosphate (Pi) that is absorbed by the intestine [7, 8]. Thus, it is commonly employed as a surrogate to estimate dietary phosphorus intake and absorption [9, 10].

However, collecting 24-hour urine is often inconvenient, and susceptible to errors in collection. The collection of 24-hour urine requires a clear definition of start and end times. Collecting either more or less urine than required can affect the results, and the sample is susceptible to external contamination during the collection period. Using formulas on phosphorus levels from spot urine samples to estimate 24hUrP may present a practical and more cost-effective alternative. However, serum Pi levels as well as renal phosphorus excretion undergo circadian changes that may affect estimates of 24hUrP from spot urine samples. Nevertheless, spot urine samples simplify scaling, reduce effort and costs, and enable wider sampling across populations. Thus, spot urine samples could serve as a viable alternative to 24-hour urine collections for estimating Pi intake. To date, different formulas have been proposed in three papers by Robinson [10], Gokce [11], and Tan [12]. Of note, all formulas were established in individuals with chronic kidney disease and eGFR <60 ml/min per 1.73 m^2^. These formulas include spot urine phosphate (uPi) excretion and factors such as age, sex, weight, and spot urine creatinine (uCr) to account for fluctuations in daily Pi excretion. It remains unclear if these formulas can be applied to individuals with normal kidney function and/or nephrolithiasis.

The aim of this study was to assess whether spot urine samples could be used to estimate 24hUrP. The specific objectives of this study were: (i) to test the accuracy of the existing equations in our cohort of individuals with eGFR >60 ml/min per 1.73 m^2^; and (ii) to develop or refine an equation to predict 24hUrP from spot urine determinations. To this end, we used data from the Swiss Kidney Stone Cohort (SKSC), which provides a large amount of clinical and biochemical data from blood and urine samples of a Swiss population comprising kidney stone formers (KSF) and non-kidney stone formers (NKSF) recruited from April 2014 to March 2020 [13].

## MATERIALS AND METHODS

### Study population

We developed equations for predicting 24hUrP using data from SKSC, a multicentric prospective observational longitudinal cohort study including 782 adult patients (age >18 years) and 207 matched controls recruited from six centers in Switzerland [13]. Patients were recruited based on their history of recurrent stones or a single stone incident coupled with at least one risk factor for recurrence. Individuals selected for the control group had no history of kidney stones and no evidence of kidney stones on a low-dose CT scan at the time of enrollment. Participants were instructed to start their 24-hour urine collection by discarding the first morning void and then to gather all urine for the next 24 consecutive hours, ending with the final void at the completion of the 24-hour period. Spot urine was collected at the second morning void, the same day after completion of the 24 h urine collections, and aliquots stored in −80°C. Participants with eGFR <60 ml/min per 1.73 m^2^ were excluded from this study. eGFR was derived using the CKD-EPI equation 2009 as the ethnic background of our cohort is mostly of European descent [14]. Registered on ClinicalTrials.gov (NCT01990027), the study adheres to the latest version of the Declaration of Helsinki, as well as ICH-GCP, GEP, and Swiss regulations on human studies. The study protocol was approved by the Swiss Cantonal Ethics Committees.

### Data

In this study, anthropometric data (BMI, age, sex), blood creatinine (Cr), Pi, albumin and uric acid, blood urea nitrogen (BUN), eGFR, 24-hour urine volume, Pi, Cr, uric acid, urea, and spot urine samples uPi and uCr data were used. To determine the 24hUrP in grams per 24 hours (g/24 h) or millimoles per 24 hours (mmol/24 h), the concentration (g/l or mmol/l) was multiplied by the total volume of the urine collected over 24 hours (ml). The urinary phosphate-to-creatinine ratio (Up/Cr) in mmol/mmol for spot urine samples was calculated with the concentrations of Pi and Cr in each sample.

### Statistical analysis

We compiled baseline data on demographics, anthropometric measurements, and kidney function in the cohort. Data were assessed for normality using quantile–quantile plots. Continuous variables are presented as either mean and standard deviation (SD) or median and interquartile range (IQR) if the data are skewed, while categorical variables are presented as percentages (%).

We developed multivariable equations to forecast 24hUrP using a two-step process. Initially, we examined the appropriate functional relationships between spot uPi and uCr concentrations and 24hUrP. For each model, we compared the transformed linear combination of spot uPi and spot uCr with the corresponding transformed spot Up/Cr. Model performance was deemed to be improved if there was a 5% or greater increase in the adjusted model *R*^2^, along with any reduction in the Akaike information criterion (AIC), a widely accepted indicator of model fit where lower values signify a better fit. Allowing visual comparisons, we produced spline smoothing and scatterplots of model-predicted versus observed 24hUrP. We applied locally estimated scatterplot smoothing regression to assess the nonlinear relationship between the predicted and observed values. In this study, a quadratic polynomial regression was used for local fitting. The smoothing was achieved using a sliding window approach, where a proportion of neighboring data points around each target point was selected for localized fitting. The span parameter, which determines the proportion of data points contributed to each localized regression estimate. The span was set to 0.75, meaning that 75% of the total data points were incorporated in each localized regression, balancing smoothness and flexibility. The degrees of freedom were controlled by employing locally linear regression to balance model flexibility and smoothness. Bland–Altman plots were used to evaluate agreement between spot uPi and 24hUrP measurements [15, 16]. The Bland–Altman plot represents the mean of predicted and observed values on the *x*-axis and the difference between predicted and observed values on the *y*-axis. A difference of zero indicates perfect agreement between the measurements. The mean difference (bias) was calculated as an indicator of systematic error, and the 95% limits of agreement (LOA) were determined as the bias ±1.96 times the SD of the differences. The bias was represented by a dashed horizontal line, and the upper and lower LOA were also plotted as dashed lines.

Next, we evaluated patient characteristics that could enhance model prediction through forward stepwise multiple linear regression with clustered variance. Based on previous studies, latent variables improving the correlation between the spot uPi and 24hUrP were considered in the multiple linear regression. Latent variables including age, sex, weight, serum Pi, serum albumin, and eGFR. We believe that BMI is a more widely used measure of body weight in clinical and epidemiological studies, as it helps reduce bias caused by individual height differences. Therefore, we chose to use BMI instead of weight as a parameter. Moreover, since our study population consist of individuals with eGFR >60 ml/min/1.72 m^2^, serum albumin levels are relatively stable with low variability, and its contribution to predicting urinary phosphate excretion may be limited. Our model is primarily based on spot uPi and uCr, along with key factors influencing urinary phosphate excretion. These variables more directly reflect the short-term dynamics of phosphate excretion and are more readily accessible in clinical settings. Therefore, we did not include serum albumin as a predictor in our model.

The covariates included in our study were age, sex, BMI, serum Pi, and eGFR. Each variable was separately introduced into a model that included the most suitable spot uPi and Cr functional form, and the variable that accounted for the largest percentage of residual variability if measured 24hUrP was retained. The correlation between 24hUrP and age, BMI, serum Pi, and eGFR was assessed using the Pearson correlation coefficient, while the correlation between 24hUrP and sex was evaluated using the Spearman correlation coefficient. Variance inflation factor (VIF) was performed on all independent variables to eliminate variables where VIF > 10. Subsequently, we individually incorporated the remaining variables and assessed whether the adjusted *R*^2^ increased by at least 5% and the AIC was reduced. This process was repeated until no additional variables met these criteria. Statistical software packages R and Empower software (http://www.empowerstats.com, X&Y Solutions, Inc., Boston, MA, USA) were used for all statistical analyses. Statistical significance was considered at a two-tailed *P* < .05.

## RESULTS

### Baseline characteristics

**Figure 1:**
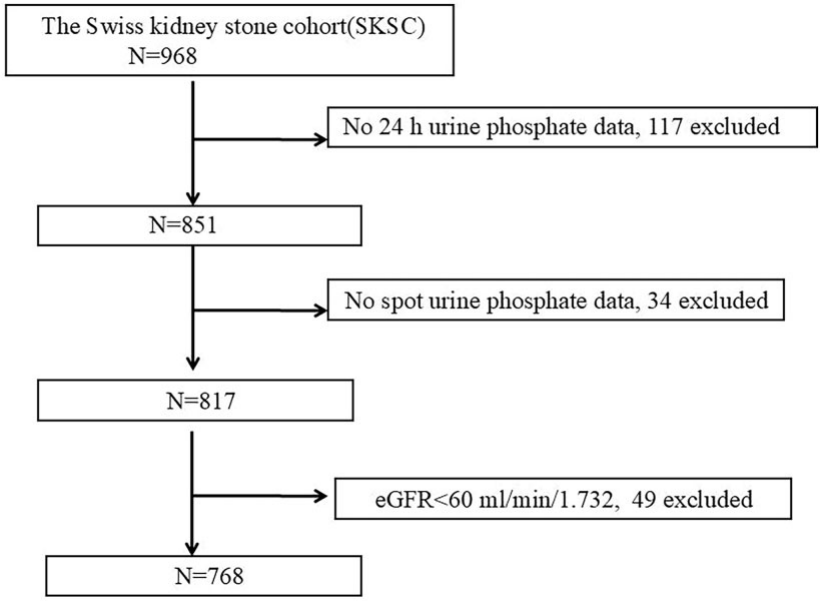
Flow chart of the study population derived from the SKSC.

Of the 968 participants enrolled in the study, we included 817 participants having both 24hUrP and spot uPi data. After excluding participants with an eGFR <60 ml/min per 1.73 m^2^, 584 kidney stone patients, and 184 healthy controls were finally included for study analysis (Fig. 1). Demographic and biochemical parameters for serum and urine of the 768 participants are shown in Table 1.

**Table 1:** Demographics and biochemical parameters for plasma and urine of study participants (*n* = 768).

	Mean ± SD or Median (Q1–Q3)		
Characteristic	Entire cohort	KSF	NKSF
height (cm)	172.35 ± 9.29	172.00 ± 9.33	173.48 ± 9.09
weight (kg)	78.91 ± 16.73	79.95 ± 16.98	75.63 ± 15.48
body mass index (kg/m^2^)	26.48 ± 4.90	26.94 ± 4.97	25.04 ± 4.36
age (years)	45.38 ± 13.87	46.34 ± 13.99	42.32 ± 13.04
	*N* (%)	*N* (%)	*N* (%)
participants	768	584	184
male	491 (63.93%)	389 (66.61%)	102 (55.43%)
Serum			
creatinine (µM)	75.00 (65.00–86.00)	75.00 (65.00–86.00)	75.00 (65.00–85.00)
BUN (mM)	4.80 (4.00–5.70)	4.80 (4.00–5.77)	4.70 (3.90–5.62)
phosphate (mmol/l)	0.99 ± 0.17	0.99 ± 0.18	1.01 ± 0.15
TMP_GFR (ml/min)	0.88 ± 0.19	0.87 ± 0.20	0.90 ± 0.16
albumin (g/l)	41.20 ± 4.42	41.70 ± 4.63	39.63 ± 3.20
uric acid (µM)	313.10 ± 77.99	317.88 ± 78.71	297.96 ± 73.84
eGFR (ml/min/1.73 m^2^)	97.65 ± 16.27	97.50 ± 16.51	98.10 ± 15.51
Urine			
urea (mmol/l)	246.87 ± 106.71	215.18 ± 102.99	195.83 ± 86.11
volume (ml)	1861.50 (1300–2410.25)	1800.00 (1230.50–2400.00)	2026.00 (1449.75–2605.00)
24-h urine phosphate (mmol/l)	14.60 (9.96–21.60)	14.91 (10.17–22.09)	13.55 (9.57–20.07)
24-h urine phosphate (mmol/day)	25.38 (19.08–33.33)	25.12 (18.50–33.20)	26.49 (20.04–34.06)
24-h urine phosphate (mg/day)	785.86 (590.92–1032.14)	777.81 (572.81–1028.07)	820.29 (620.79–1054.99)
creatinine (µmol/l)	6908.50 (4781.25–9900.00)	7174.00 (4961.00–10306.00)	6353.50 (4572.00–8945.25)
uric acid (µmol/l)	1670.00 (1160.00–2379.50)	1702.00 (1195.50–2440.00)	1532.50 (1123.00–2063.75)
spot urine phosphate (mmol/l)	14.10 (7.66–22.97)	14.40 (7.82–22.98)	12.42 (6.38–22.92)
spot urine creatinine (mmol/l)	10.19 (6.86–15.58)	10.27 (7.04–15.53)	10.02 (5.54–15.74)
spot phosphate/creatinine ratio	1.33 (0.93–1.90)	1.37 (0.96–1.94)	1.27 (0.90–1.73)

First, we used the three previously reported prediction models to estimate the 24hUrP from spot urine samples in our study cohort (details shown in Table 2) [10–12]. In two published models the participants had an older mean age and lower eGFR compared to our study cohort [10, 12], while the patients used for the model proposed by Gokce had a similar mean age to ours, with no eGFR information but 20% of the participants being considered healthy [11]. Smooth curve fitting and Bland–Altman diagrams were plotted for each equation, allowing visual comparisons. As shown in Supplementary Fig. 1, the formula developed by Robinson showed the best performance of the three equations [10], yet Bland–Altman analysis of predicted 24hUrP concentrations derived from spot urine samples showed a positive bias of 0.51 mg/day (95% CI −0.32 to 1.34), compared to the observed 24hUrP concentration (Supplementary Fig. 1B). Thus, we conclude that these three published formulas cannot accurately predict 24hUrP in our study cohort.

**Table 2:** Equations to estimate 24-h phosphate excretion from urine spot sample.

Reference	Description	Formula to estimate 24-h phosphate excretion
Robinson *et al.*	Developed with a sample of 143 patients, the study had a mean age of 66 ± 13, an eGFR ranging from 20 and 45 ml/min per 1.73 m^2^, and included 46.85% male participants	e^(6.7 + 0.44^ ^×^ ^(Ln(Up/Cr^^)) −^ ^006*(age) + 0.005^ ^× (^^weight, kg) + 0.30 if male)^, mg/day
Gokce *et al.*	Developed with a sample of 67 participants (aged 16 to 85 years; 49.25% participants were male; weight, 35 to 100 kg) prospectively from the patient population of Firat University Hospital, Elazig, Turkey	(Up/cr + 0.012)/0.975, g/day
Tan *et al.*	Developed with 116 participants, 77 CKD patients (aged 55 to 74 years, 74% were male, eGFR ranging from 26.8 and 50.1 ml/minute per 1.73 m^2^) and 39 healthy volunteers (aged 36 to 57, 43.6% were male, eGFR ranging from 95 and 112.4 ml/minute per 1.73 m^2^) at The Royal Melbourne Hospital	e ^(2.794 + (0.213 × (Ln(spotuPi))) − (1.161 × serum phosphate)^^+ (0.027 × serum albumin) + (0.002 × eGFR))^, mmol/day

### Model development

**Figure 2:**
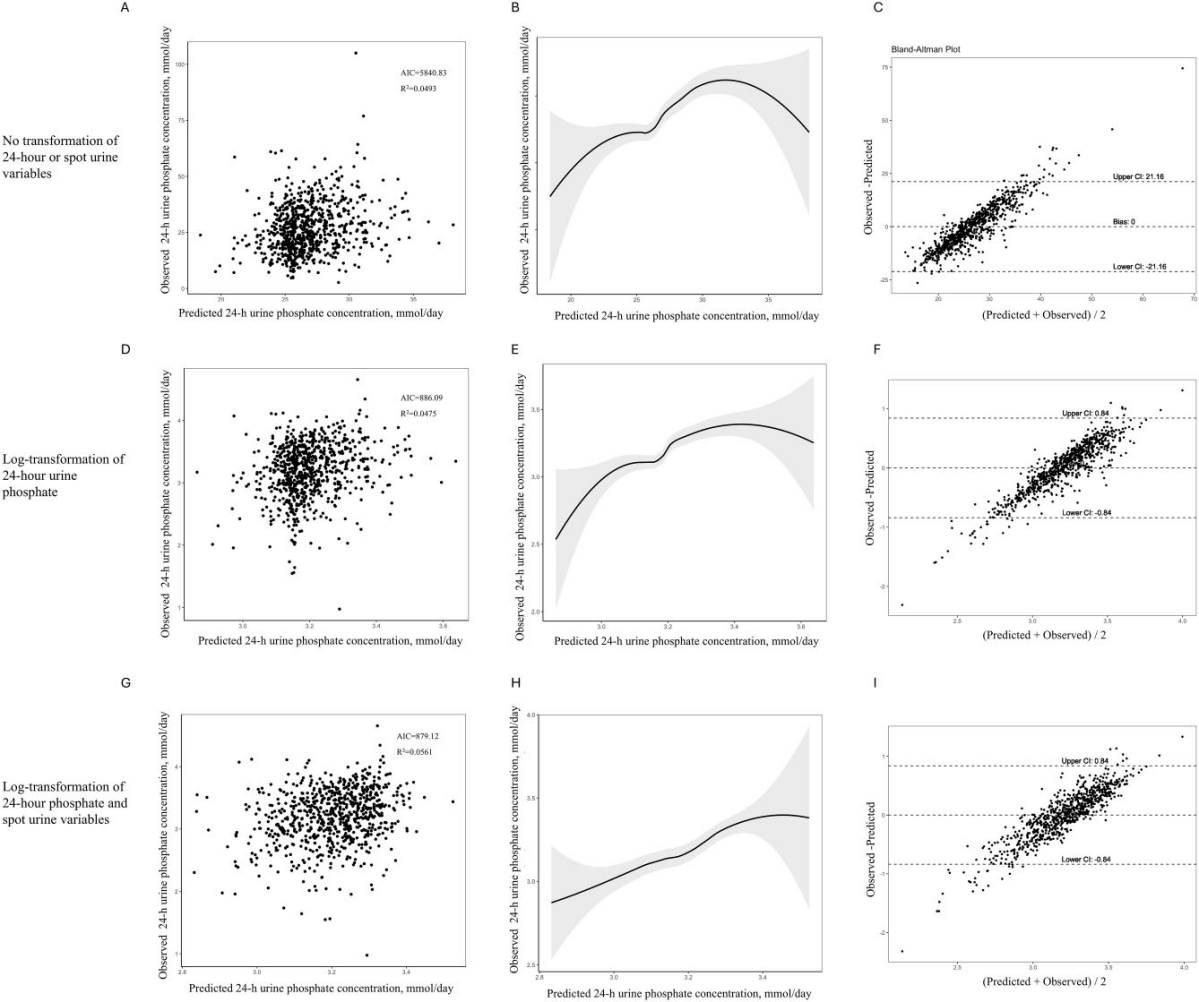
Correlations of measured versus predicted 24-h urine phosphate for linear combination of spot urine phosphate and urine creatinine concentrations. (**a, d, g**) Scatterplots of measured 24-h phosphate excretion compared with 24-h phosphate excretion estimated from urine spot samples. (**b, e, h**) The nonlinear relationship between predicted and observed 24-h urine phosphate concentration. A smooth curve fitting between variables is represented by the solid black line. The 95% confidence interval of the fit is indicated by the gray line. (**c, f, i**) Bland–Altman plots of the average of 24-h phosphate excretion versus the difference between the estimated 24-h phosphate excretion from urine spot samples and the measured 24-h phosphate excretion. The *x*-axis indicates the mean of measured and estimated 24-h phosphate excretion; the *y*-axis indicates the mean difference between estimated and measured 24-h phosphate excretion; the continuous lines indicate the mean differences; and the dashed lines indicate the 95% LOA of the mean difference (mean ± 1.96 SD).

**Figure 3:**
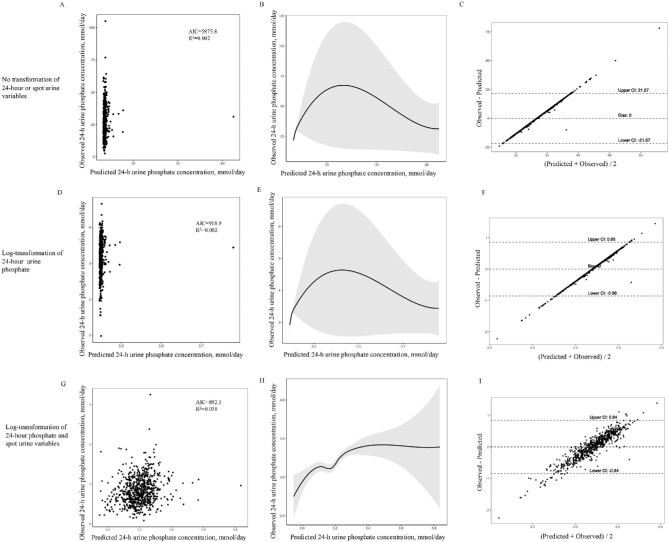
Correlations of measured versus predicted 24-h urine phosphate for spot urine phosphate-to-creatinine ratio using no transformation, partial or complete log transformation. (**a, d, g**) Scatterplots of measured 24-h phosphate excretion compared with 24-h phosphate excretion estimated from urine spot samples. (**b, e, h**) The nonlinear relationship between predicted and observed 24-h urine phosphate concentration. A smooth curve fitting between variables is represented by the solid black line. The 95% confidence interval of the fit is indicated by the gray line. (**c, f, i**) Bland–Altman plots of the average of 24-h phosphate excretion versus the difference between the estimated 24-h phosphate excretion from urine spot samples and the measured 24-h phosphate excretion. The *x*-axis indicates the mean of measured and estimated 24-h phosphate excretion; the *y*-axis indicates the mean difference between estimated and measured 24-h phosphate excretion; the continuous lines indicate the mean differences; and the dashed lines indicate the 95% LOA of the mean difference (mean ± 1.96 SD).

In each of the three functional models developed and evaluated—the untransformed model, the model with log-transformed 24hUrP only, and the model with log-transformed 24hUrP and spot urine measurements—the combination of spot uPi and uCr demonstrated superior performance compared to the spot Up/Cr, as shown in Figs. 2 and 3 in the Bland–Altman plots illustrating the mean biases of the models. The best fitting model was log-transformed 24hUrP with the log-transformed spot urine measurements (model *R*^2^ = 0.0561, AIC = 879.12), with a bias of −0.00 mmol/day (95% CI −0.85 to 0.85). The functional form was retained and applied later in forward stepwise regression models. Parameters showing a statistically significant univariate correlation with 24hUrP included BMI, sex, age, serum Pi, and eGFR.

Based on the *R*² and AIC criteria, BMI, sex, and age were recognized as independent predictors of 24hUrP, along with spot uCr and spot uPi (Table 3). Serum Pi and eGFR did not increase the predictive accuracy of the model, as the increase in the *R*^2^ value after incorporating these parameters did not exceed 5%. Based on this, the final prediction equation is derived as follows: Ln(24hUrP) = 2.6417 + 0.0146 × BMI + 0.1969 × Ln( spot uPi) − 0.16605 × Ln(spot uCr) − 0.0032 × age + 0.3 (if male).

**Table 3:** Independent predictors of log 24-h urine phosphate beyond the spot urine phosphate and urine creatinine.

	β	95%CI	*P*	Incremental *R*^2^	Decremental AIC
(intercept)	2.6417	2.45, 2.83	<.0001		
male gender	0.3000	0.24, 0.36	<.0001	0.0936	845.9795
Ln (spot phosphate)	0.1969	0.15, 0.25	<.0001	0.1198	825.4630
Ln (spot creatinine)	−0.1660	−0.22, −0.11	<.0001	0.1527	798.1956
BMI	0.0146	0.01, 0.02	<.0001	0.1728	781.7933
age	−0.0032	−0.01, −0.00	.0035	0.1820	775.1857

**Figure 4:**
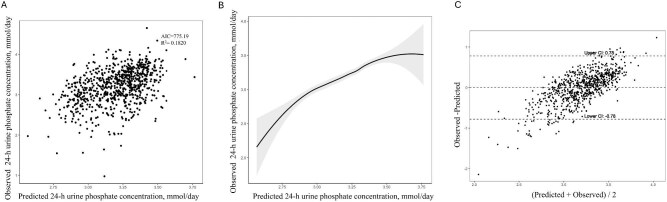
Correlation of predicted and measured 24 h urine phosphate excretion using the developed formula. (**a**) Scatter plots of measured 24-h phosphate excretion compared with 24-h phosphate excretion estimated from urine spot samples. (**b**) The nonlinear relationship between predicted and observed 24-h urine phosphate concentration. A smooth curve fitting between variables is represented by the solid black line. The 95% confidence interval of the fit is indicated by the gray line. (**c**) Bland–Altman plots of the average of 24-h phosphate excretion versus the difference between the estimated 24-h phosphate excretion from urine spot samples and the measured 24-h phosphate excretion.

**Figure 5:**
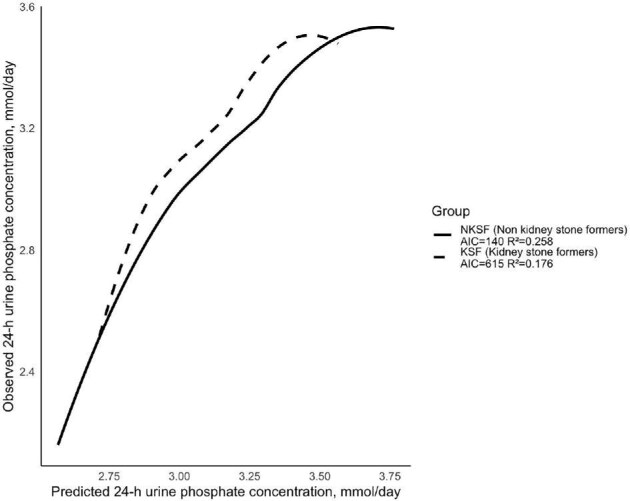
The nonlinear relationship between predicted and observed 24-h urine phosphate amount in the groups of KSF (dashed line) and NKSF (solid line).

The final 24hUrP prediction model that provided the best *R*^2^ value explained an estimated 18.2% of variation in 24hUrP (*R*^2^ = 0.182, AIC = 775.19, Fig. 4). Last, we categorized the research participants into groups of patients with kidney stones and individuals without kidney stones to determine how the formula would perform in these two populations. The smooth curve fitting indicates that this formula is slightly better suited for the NKSF (Fig. 5). We compared the urinary relative supersaturation (RS) of calcium phosphate (CaP) between the two groups and we found that KSF had a higher RS of CaP than NKSF (Supplementary Fig. 2). Pearson correlation analysis showed that RS versus difference between predicted and observed 24hUrP has a weak positive correlation in both groups, although the relationship is more significant in KSF (*r* = 0.186, *P* < .001).

## DISCUSSION

The current study involved 584 KSF and 184 NKSF as assessed by low-CT scans at the time of recruitment from the SKSC. Initially, we assessed the accuracy of three previously published predictive formulas for estimating 24hUrP using spot urine samples. Smooth curve fitting and Bland–Altman diagrams indicated that the formula from Robinson *et al.* showed the best performance in estimating 24hUrP in our cohort but with considerable bias. Second, to develop a more accurate prediction formula, a new predictive equation for estimating 24hUrP excretion using spot uPi and uCr levels, along with BMI, sex, and age was developed by a stepwise multivariate linear regression approach. This new formula represents an advance, providing a more reliable estimation of 24hUrP excretion based on spot Pi and Cr data suitable for both clinical application and further studies in individuals with a kidney function as assessed by eGFR >60 ml/min/1.73 m^2^. By comparing the RS of CaP between KSF and NKSF, we found that kidney stone patients have a higher level of RS of CaP. This may indicate a higher propensity to form insoluble CaP crystals that are lost during the initial centrifugation steps before measuring phosphate levels and thereby may lead to an underestimation of 24hUrP, which could explain why the formula is more applicable to the non-kidney stone group.

The correlation between spot uPi levels and 24hUrP excretion has been previously examined. Most recently, this relationship was examined through a single center observational cross-section study with 116 participants (77 with kidney disease and 39 healthy volunteers at The Royal Melbourne Hospital) [12]. This study reported median spot Up/Cr and 24hUrP excretion with 1.7 (1.3–2.2) mmol/mmol and 25.8 (19.9–35.0) mmol/day, which is close to our median spot Up/Cr 1.33 (0.93–1.90) mmol/mmol and 24hUrP excretion 25.38 (19.08–33.33) mmol/day. However, no significant relationship between spot Up/Cr and 24hUrP excretion was found with a low correlation coefficient of 0.064 (*P* = .51). By contrast, a correlation was observed between spot uPi and 24hUrP (*R* = 0.385, *P* < .001). The 24hUrP prediction model incorporated independent variables such as eGFR, serum Pi, albumin, and spot uPi. Conversely, in an earlier study of 143 patients with an eGFR between 20 and 45 ml/minute per 1.73 m^2^ from the Phosphate Normalization Trial [10], a novel equation was described to predict 24hUrP excretion using spot uPi and Cr, age, sex, and body weight. In our cohort, this equation proved to be both more accurate compared to using the Up/Cr alone. It offers an easy way to estimate 24hUrP excretion in patients with CKD but not on dialysis. Additionally, the authors validated the effectiveness of the developed model by applying it to a distinct random sample of 70 patients with non-dialysis requiring CKD from a separate observational study. A third study conducted by Gokce *et al.* among 67 participants from the patient population of Firat University Hospital, Elazig, Turkey, displayed strong correlations between the spot urine calcium-to-creatine ratio and 24-hour total calcium excretion (*R*^2^ = 0.90) and between the spot Up/Cr and 24hUrP (*R*^2^ = 0.93) [11]. These correlations were consistent regardless of factors such as gender; renal function status; varying levels of serum calcium, Pi, and parathyroid hormone; and the participants’ mobility. Nevertheless, the regression model conducted in this study was constrained to pass through the origin, without considering the goodness of fit, which would potentially over-estimate the value of *R*^2^.

In nutritional research, metabolic balance studies, and assessment of urinary risk factors for stone formation [17–19], the measurement of 24hUrP is used. However, collecting urine over a 24-hour period may be cumbersome for patients and more expensive than providing spot urines samples. Also, incomplete sampling can influence results. Our data suggest that spot urine samples may provide a reliable and simpler source to estimate 24-hour urine phosphorus excretion. Although our predictive equation provided a slightly better prediction for NKSF, its implications may extend beyond this specific population, particular in understanding phosphate handing in KSF and its potential relevance to early kidney disease. Phosphate homeostasis plays a crucial role in kidney health, and its dysregulation is a key factor in CKD progression. While our study focuses on individuals with eGFR >60 ml/min per 1.73 m^2^, the observed variations in urinary phosphate excretion among KSF and NKSF may potentially suggest differences in renal phosphate handing. Notably, the higher RS of CaP in KSF may reflect a predisposition to phosphate retention or altered phosphate transport mechanisms. These finding raise the possibility that our equation could provide insights into early abnormalities of phosphate metabolism.

The main strength of our study is that Pi excretion was measured using 24-hour urine collection and spot samples in 768 participants, which to our knowledge is a larger number of participants than any previous study that predicted 24hUrP. Moreover, data were obtained from participants with eGFR >60 ml/min/1.73 m^2^ making it widely applicable. However, there were several limitations to this study. We only collected the second morning spot sample to estimate 24hUrP. Further studies will be needed to evaluate the circadian dependency and thus the conversion factor of different time points during the day. Moreover, we did not have a validation cohort and our cohort is predominantly of European descent.

In conclusion, our data verified that 24hUrP is reflected by spot Pi and Cr levels, while the addition of commonly and simply available clinical data of BMI, sex, and age enhances the predictive accuracy and allows for a simple and inexpensive prediction that can be integrated into clinical routine.

## Supplementary Material

sfaf097_Supplemental_File

## Data Availability

The data underlying this article will be shared on reasonable request to the corresponding author.
