# Supplementary material for: Estimating 24-hour urine phosphate excretion from spot urine
Source: Clin Kidney J. 2025 Apr 10;18(5):sfaf097. doi: 10.1093/ckj/sfaf097 (PMC12067064; doi:10.1093/ckj/sfaf097)
Supplement: sfaf097_Supplemental_File [file sfaf097_supplemental_file.docx]

**SUPPLEMENTARY MATERIAL**

**Li et al. Estimating 24-Hour Urine Phosphate Excretion from Spot Urine**

**
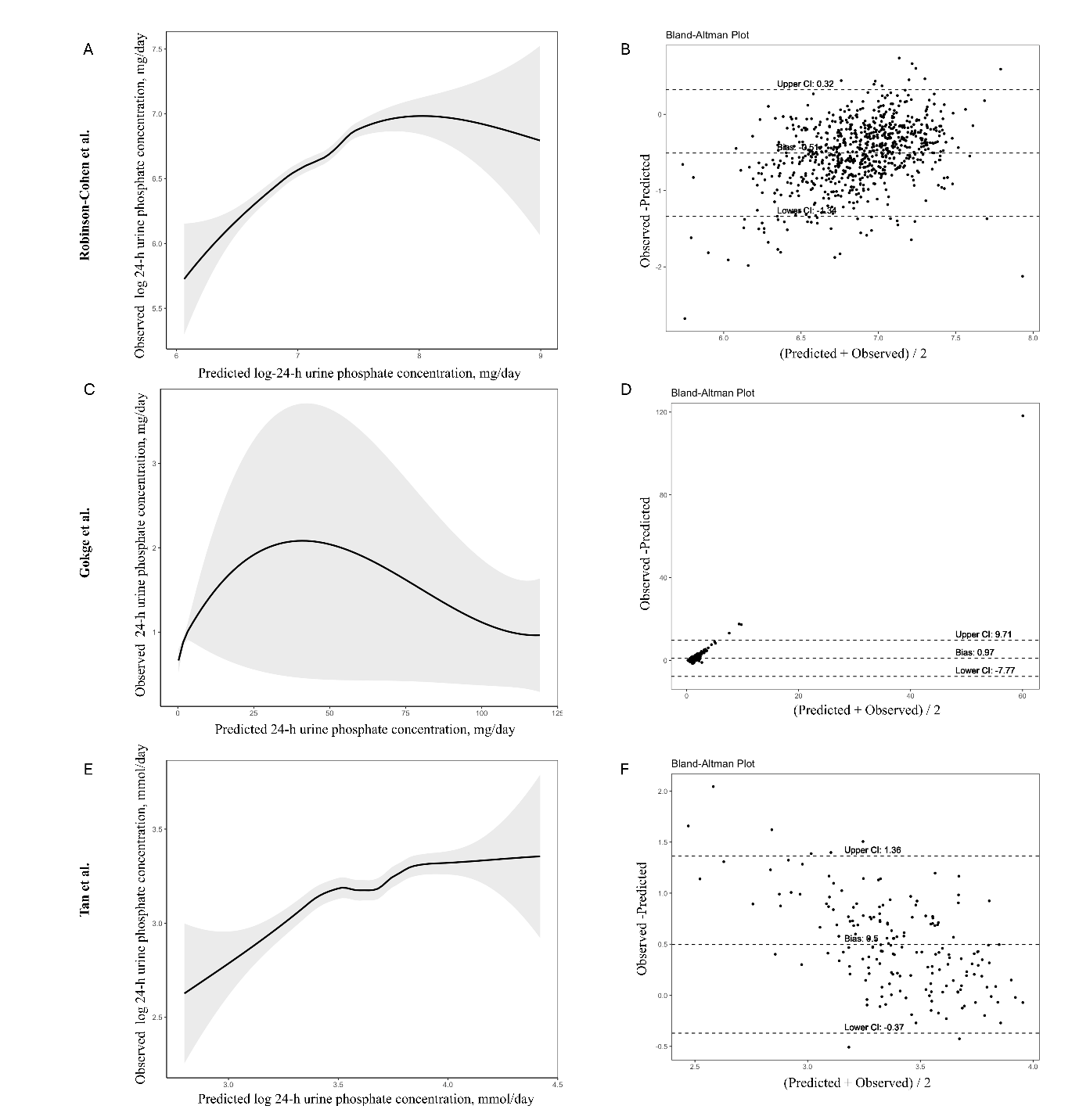
**

**Supplementary figure 1**

Smooth curve fitting and Bland-Altman diagrams for the three previously published equations to predict 24 h urine phosphate excretion from spot urine [1-3].


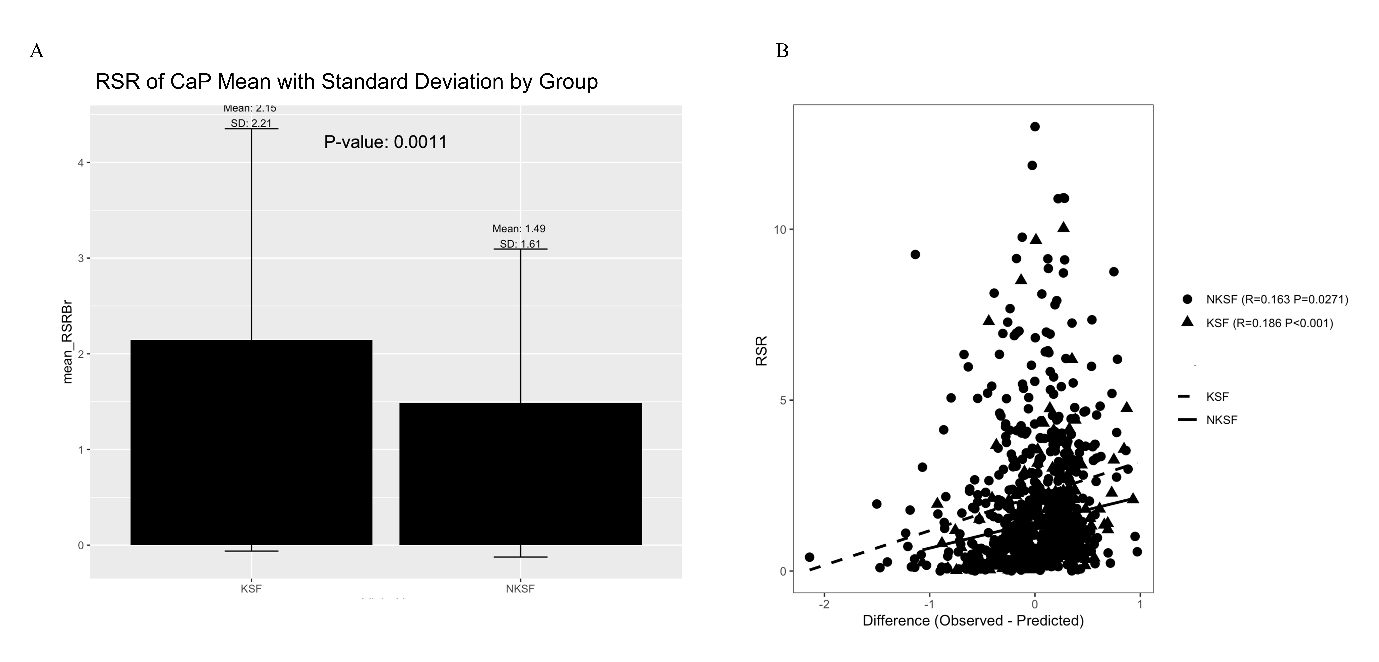


**Supplementary figure 2**

The differences between KSF and NKSF in relative supersaturations (RS) of calcium phosphate (CaP) and the correlation of RS with the difference between predicted and observed 24hUrP.

**References**

1. Robinson-Cohen C, Ix JH, Smits G, et al. Estimation of 24-hour urine phosphate excretion from spot urine collection: development of a predictive equation. *J Ren Nutr*. May 2014;24(3):194-9. doi:10.1053/j.jrn.2014.02.001

2. Gokce C, Gokce O, Baydinc C, et al. Use of random urine samples to estimate total urinary calcium and phosphate excretion. *Arch Intern Med*. Aug 1991;151(8):1587-8. doi:10.1001/archinte.1991.00400080083015

3. Tan SJ, Smith ER, Cai MM, Holt SG, Hewitson TD, Toussaint ND. Relationship between timed and spot urine collections for measuring phosphate excretion. *Int Urol Nephrol*. Jan 2016;48(1):115-24. doi:10.1007/s11255-015-1149-z
